# Quantifying the Impact of Scenic Environments on Health

**DOI:** 10.1038/srep16899

**Published:** 2015-11-25

**Authors:** Chanuki Illushka Seresinhe, Tobias Preis, Helen Susannah Moat

**Affiliations:** 1 Data Science Lab, Behavioural Science, Warwick Business School, University of Warwick, Coventry, CV4 7AL, UK

## Abstract

Few people would deny an intuitive sense of increased wellbeing when spending time in beautiful locations. Here, we ask: can we quantify the relationship between environmental aesthetics and human health? We draw on data from *Scenic-Or-Not*, a website that crowdsources ratings of “scenicness” for geotagged photographs across Great Britain, in combination with data on citizen-reported health from the Census for England and Wales. We find that inhabitants of more scenic environments report better health, across urban, suburban and rural areas, even when taking core socioeconomic indicators of deprivation into account, such as income, employment and access to services. Our results provide evidence in line with the striking hypothesis that the aesthetics of the environment may have quantifiable consequences for our wellbeing.

Few people would deny that spending time in areas of beautiful scenery results in a sense of increased wellbeing. Yet, what if scenic environments had an impact on our health? While the question of how the aesthetics of the environment might relate to our wellbeing has been of interest to researchers for many years, studies to date have been limited to investigations of differences in reactions to urban and natural scenes^1^,^2^,^3^,^4^,^5^,^6^,^7^,^8^,^9^, research into the role of greenspace and vegetation in urban environments^7^,^10^,^11^,^12^,^13^,^14^,^15^,^16^,^17^,^18^,^19^,^20^, or analyses of small-scale survey data^21^,^22^,^23^ due to the impracticality of gathering large-scale data on humans’ perception of the environment.

The ubiquitous presence of the Internet in today’s society, however, has led to the creation of a new source of information on human behavior: large datasets of online activity. Analysis of data obtained from platforms such as *Google*, *Flickr*, *Wikipedia* and *Twitter* has already led to a range of new insights into human behavior in the real world^24^,^25^,^26^,^27^,^28^,^29^,^30^,^31^,^32^,^33^,^34^,^35^,^36^,^37^.

Here, we use data from *Scenic-Or-Not*^38^, a website that crowdsources ratings of “scenicness,” in order to develop a better understanding of how the aesthetics of the environment may impact our health. *Scenic-Or-Not* users rate random geotagged photographs of Great Britain on an integer scale of 1–10, where 10 indicates “very scenic” and 1 indicates “not scenic”. The *Scenic-Or-Not* website comprises 217,000 images covering nearly 95% of the 1 km grid squares of Great Britain. As of August 2014, the *Scenic-Or-Not* dataset contained 1.5 million votes.

We first explore the nature of photographs rated as scenic, including their color composition. Next, we evaluate to what degree scenicness relates to an objective measurement, green land cover, as several studies analyzing green land cover data indicate that an abundance of greenspace results in increased human wellbeing^11^,^12^,^13^,^14^,^15^,^16^,^17^,^18^,^19^,^20^.

Finally, we investigate whether scenicness can explain more of the variance in geographic self-reports of health than data on green land cover are able to alone. In previous studies, self-reported health has been shown to be a strong predictor of subsequent mortality rates^39^,^40^.

We analyze this relationship across urban, suburban and rural areas, to take into account that the definition of scenicness may vary depending on environmental context. As less scenic areas may also be areas of higher air pollution, we run an additional analysis including modeled estimates of concentrations of the following pollutants: sulphur dioxide (SO_2_), oxides of nitrogen (NOx), particles and fine particles (PM_10_ and PM_2.5_), benzene (C_6_H_6_), carbon monoxide (CO) and ozone (O_3_)^41^.

## Results

**Figure 1:**
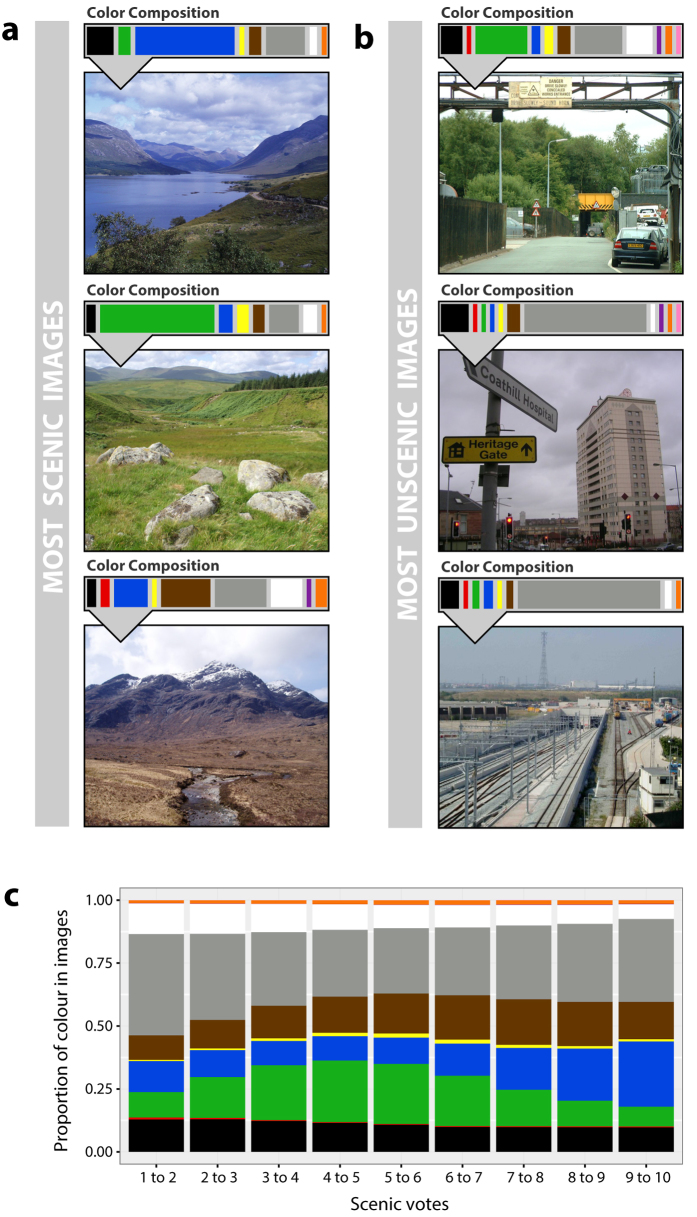
The color composition of scenic and unscenic images from *Scenic-Or-Not*. (**a**) A sample of the most scenic images reveals that they not only contain large areas of greenspace but also large proportions of grey, brown and blue. These may be mountainous landscapes or water features. (**b**) A sample of the least scenic images shows that “unscenic” images can also contain green, but the presence of manmade objects may be affecting the rating. Photographers of scenic images from top to bottom: Jamie Campbell (http://www.geograph.org.uk/photo/9007), Peter Standing (http://www.geograph.org.uk/photo/211685), David Gruar (http://www.geograph.org.uk/photo/158649). Photographers of unscenic images from top to bottom: David Hignett (http://www.geograph.org.uk/photo/35895), Chris Upson (http://www.geograph.org.uk/photo/142605), Glyn Baker (http://www.geograph.org.uk/photo/48959). Copyright of the images is retained by the photographers. Images are licensed for reuse under the Creative Commons Attribution-Share Alike 2.0 Generic License. To view a copy of this license, visit http://creativecommons.org/licenses/by-sa/2.0/ (**c**) We analyze the average color composition in images of varying scenicness ratings. While one may expect the proportion of green in images to increase as scenicness ratings increase, we find instead that images rated highly for scenicness tend to have a high proportion of blue, brown and grey. Less scenic images tend to be mainly grey with higher proportions of black and white, but also contain more green pixels than the images rated highly for scenicness.

Examining a sample of *Scenic-Or-Not* photographs helps us understand what constitutes scenicness (Fig. 1). Visual inspection of photographs with the highest rating for scenicness reveals that they tend to contain landscapes with broad open areas and essentially no manmade structures (Fig. 1A). Unscenic photographs appear to contain mostly manmade structures including roads, buildings and cars (Fig. 1B). Crucially, we observe that even unscenic images can have large areas of greenspace, though often with manmade structures that obstruct the view of the greenspace. The existence of such photographs provides initial evidence that the presence of green in an image is not sufficient for it to be considered scenic.

We therefore assess how perceived colors, in general, correspond with objective reports of the environment. We examine each image from *Scenic-Or-Not* on a per-pixel level, with each pixel being allocated to one of eleven colors that constitute principal colors in the English vocabulary (black, blue, brown, grey, green, orange, pink, purple, red, white, yellow). As color naming varies from one individual to another^42^, we draw on crowdsourced data generated through an online survey of 1.5 million participants to determine to which color a pixel should be allocated. More details of this procedure can be found in the Supplementary Information.

Figure 1C depicts the relationship between scenicness ratings and the proportion of each color found in the images. Although all colors are significantly correlated with scenicness (all |*τ* |*s* > 0.01, all *ps* < 0.001, *N* = 206,873, Kendall’s rank correlation), Fig. 1C suggests that the association between color and scenicness is complex. While one may expect scenicness ratings to steadily increase as the proportion of green in images increases, visual inspection of the data instead reveals that highly scenic images tend to have a high proportion of blue, grey and brown. This may be due to open skies and mountains in highly-rated images. Less scenic images tend to be mainly grey with higher proportions of black and white, but also more green pixels than the highly-rated scenic images.

Scenicness therefore does not appear to constitute a simple predominance of green areas. In order to further explore how scenicness compares to greenspace, we compare green land cover, as reported in the *Generalised Land Use Database Statistics for England 2005*^43^, to the scenicness ratings extracted from *Scenic-Or-Not*. The *Generalised Land Use Database* covers all land in England. We analyze land use data describing England at the level of Lower Layer Super Output Areas (LSOA), which are geographic areas with an average population size of 1,600, defined by the Office of National Statistics for statistical analyses, with areas ranging between 0.018 square km to 684 square km. To combine the land use data with the *Scenic-Or-Not* ratings, which are provided as ratings for one geotagged photograph in each 1 km grid square, we calculate the average scenic rating of all *Scenic-or-Not* photographs taken within each LSOA. Using this method, scenicness ratings are available for 16,907 out of all 32,844 English LSOAs.

**Figure 2:**
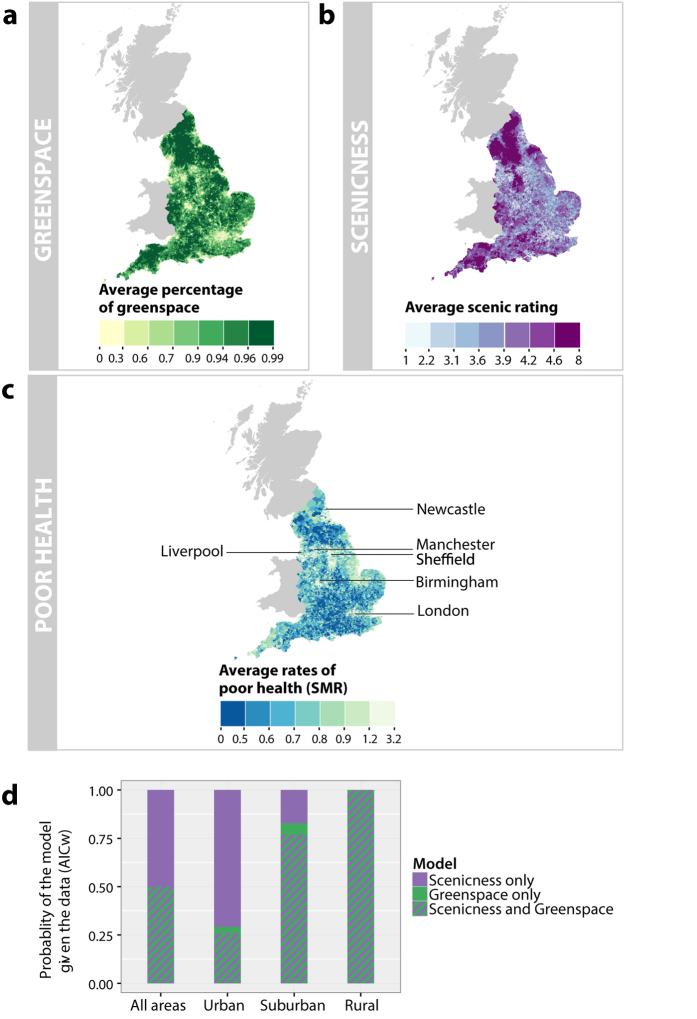
Scenicness, greenspace and health in England. (**a**) Previous studies have suggested that greater amounts of greenspace are associated with reports of better health. We depict greenspace, utilizing *Generalised Land Use Database 2005* green land cover data, at the level of English Lower Layer Super Output Areas (LSOAs) with quantile breaks. (**b**) We investigate how scenicness compares to greenspace, as scenicness and green land cover are significantly correlated (*τ* = 0.2, *p* < 0.001, *N* = 128,213, Kendall’s rank correlation). We calculate the average scenic rating of all *Scenic-Or-Not* photographs taken for each LSOA and depict these ratings using quantile breaks. Visual inspection of maps A and B reveals that, while the two measures are significantly correlated, there appear to be differences, for example in the East of England. (**c**) Respondents to the *2011 Census for England and Wales* classified their health as “Very good or good”, “Fair” or “Bad or very bad”. We calculate health rates using the Standardized Morbidity Ratio (SMR), which is the ratio of the observed to the expected number of cases of bad health for a particular population, taking the age and gender of inhabitants into account. We depict the SMR for each LSOA using quantile breaks. (**d**) We investigate to what extent geographic differences in health can be explained by scenicness and greenspace, by creating Conditional Autoregressive (CAR) models where we also control for socioeconomic deprivation using data from the *2010 English Indices of Deprivation.* To determine which model provides the best fit for predicting poor health, we calculate Akaike weights (AICw), which can be used to interpret the probability of each model given the data. Details on how AICw are calculated can be found in the Methods section. In all cases, we find that there is more evidence for models that include scenicness (denoted by purple or by purple and green stripes) than for the model with only greenspace (denoted by green). Maps created using the R packages rgdal and ggplot2. Contains National Statistics, NISRA, NRS and Ordnance Survey data © Crown copyright and database right 2013.

We find that scenicness and green land cover are significantly correlated (*τ* = 0.2, *p* < 0.001, *N* = 128,213, Kendall’s rank correlation). The relationship between scenicness (Fig. 2A) and green land cover (Fig. 2B) is apparent upon inspection of the two maps. However, the correlation is not very strong in terms of effect size, suggesting that scenicness and green land cover are not necessarily the same. For example, in the East of England, green land cover and scenicness diverge considerably. We therefore investigate to what extent these two different variables can help us understand geographic differences in health.

We draw on geographic data from the *2011 Census for England and Wales* capturing respondents’ classification of their health as “Very good or good”, “Fair” or “Bad or very bad”^44^. Following Mitchell and Popham^14^, we calculate health rates using the Standardized Morbidity Ratio (SMR), which is the ratio of the observed to the expected number of cases of bad health for a particular population, taking the age and gender of inhabitants into account. Following standard practice in other studies^13^,^14^,^15^,^16^,^17^, we control for socioeconomic characteristics that may be linked with reports of health. We use deprivation data from the relevant domains of the 2010 English Indices of Deprivation^45^: Income Deprivation, Employment Deprivation, Education Skills and Training Deprivation, Barriers to Housing and Services, Living Environment Deprivation, and Crime. The value of these indices increases in line with the proportion of people who experience deprivation in each domain.

Finally, in order to explore whether there is any variation in the association between health and scenicness across urban, suburban and rural areas, we use the 2011 Rural-Urban Classification^46^. For the purposes of this study, “urban” is defined using the category “Urban Major Conurbation” from the 2011 Rural-Urban Classification. The remaining urban categories are deemed suburban. Scenicness data is available for 3,945 urban LSOAs, 7,781 suburban LSOAs, and 5,182 rural LSOAs.

We investigate to what extent geographic differences in self-reported health, as measured using the Standard Morbidity Ratio (SMR), can be explained by scenicness and greenspace. To carry out this analysis, we build a Conditional Auto Regressive (CAR) model, which takes spatial autocorrelation into account. As with time series data, where observations that are closer in time may be correlated and hence violate the linear regression assumption that observations are independent, spatial data may also exhibit autocorrelation where neighboring areas may be more or less alike^47^.

We confirm the need for this approach by initially building two different linear regression models to predict poor reports of health at the level of LSOAs. Both models include the socioeconomic variables describing estimates of deprivation across income, employment, education, housing, crime and living conditions. The first model additionally includes scenicness only, while the second model additionally includes greenspace only. We then run a Moran’s I test on the residuals of both models to test for spatial auto-correlation. Both models exhibit significant spatial autocorrelation in the residuals of the linear regression models (Scenic model: *Moran’s I* = 0.143, *p* < 0.001, *N* = 16,907; Greenspace model: *Moran’s I* = 0.136, *p* < 0.001, *N* = 16,907).

We therefore investigate to what extent geographic differences in reports of health can be explained by scenicness and greenspace, by running three different CAR models. Again, all models include the socioeconomic variables mentioned above. The first CAR model additionally includes both scenicness and greenspace. However, as our previous correlation analysis indicates that scenicness is significantly correlated with greenspace (*τ* = 0.2, *p* < 0.001, *N* = 128,213), we run two additional CAR models: one that includes greenspace only, and a second that includes scenicness only.

Table 1 provides results of the CAR model that includes both scenicness and greenspace. Across the entire English dataset, we find that lower values of scenicness are significantly associated with reports of worse health (*β* = −0.008, *p* < 0.001, *N* = 16,907), even when taking a wide range of deprivation variables into account. We also find that this relationship holds across urban, suburban and rural areas (Urban: *β* = −0.007, *p* = 0.012, *N* = 3,944, Suburban: *β* = −0.005, *p = *0.007, *N* = 7,781, Rural: *β* = −0.012, *p* < 0.001, *N* = 5,182). However, in this model, while greenspace is not associated with reports of poor health in general (all *p*s > 0.22 for urban, suburban and rural areas as well as England as a whole), more greenspace is significantly correlated with reports of poor health in suburban areas (*β* = −0.020, *p* = 0.024, *N* = 7,781).

In the second CAR model (Table 2) with scenicness removed, less greenspace is significantly correlated with reports of worse health when considering England as a whole (*β* = −0.019, *p* = 0.003, *N* = 16,907). However, this effect is not significant for urban areas, suburban areas or rural areas when considered separately.

In the third CAR model (Table 3), with greenspace removed, the results are similar to the first model that includes both scenicness and greenspace. Lower ratings of scenicness are significantly associated with reports of worse health across the entire English dataset (*β* = −0.008, *p* < 0.001, *N* = 16,907), as well as urban, suburban and rural areas when considered individually (Urban: *β* = −0.009, *p* = 0.010, *N* = 3,944, Suburban: *β* = −0.004, *p* = 0.028, *N* = 7,781, Rural: *β* = −0.011, *p* < 0.001, *N* = 5,182).

Finally, in order to determine which of the three models provides the best fit for predicting reports of poor health, we rank all three models in terms of their Akaike Information Criterion (AIC) values. This provides a measure of the likelihood of a given model and its free parameters. In order to compare the fit of the models to each other, AIC values are then transformed to Akaike weights (AICw) following the method proposed by Wagenmakers and Farrel^48^. These weights can be interpreted as the probability of each model, given the data. This model comparison indicates that models including scenicness have more explanatory power than the model with only greenspace (Fig. 2D).

One final concern could be that less scenic areas may also be areas of higher pollution, which could impact the health of local residents. Our examination of water pollution data held by the World Bank suggests that 100% of the United Kingdom population has access to an “improved water source” (defined as water that has been modified or protected from outside contamination)^49^. Furthermore, in accordance with the guidelines specified by the EU Drinking Water Directive for England and Wales, the Drinking Water Inspectorate (the independent regulator of public drinking water supplies in England and Wales) reports that only 0.04% of the 1.9 million tests conducted in 2011 failed to meet one of the chemical or microbiological standards^50^. Due to the overwhelmingly high level of water quality in the UK, we therefore conclude that further analysis of water pollution is unwarranted.

In our original model, a measure of air pollution is included in the Living Environment Deprivation variable. However, as air pollution is an ongoing health concern, particularly in urban areas, we run a further analysis including modeled estimates of concentrations of the following pollutants: sulphur dioxide (SO_2_), oxides of nitrogen (NOx), particles and fine particles (PM_10_ and PM_2.5_), benzene (C_6_H_6_), carbon monoxide (CO) and ozone (O_3_). Air quality data at a 1 km^2^ resolution was obtained from the UK Air Information Resource for the year 2011.

We build a CAR model that includes both scenicness and greenspace, as well as the air pollutant variables (Table S2). Even after explicitly including the air pollutant measurements, we continue to find that lower scenicness is significantly associated with reports of worse health across the entire English dataset (*β* = −0.006, *p* < 0.001, *N* = 16,907) as well as urban, suburban and rural areas when considered individually (Urban: *β* = −0.008, *p* = 0.010, *N* = 3,944, Suburban: *β* = −0.004, *p* = 0.0259, *N* = 7,781, Rural: *β* = −0.008, *p = *0.007, *N* = 5,182).

We also rank three models in terms of their Akaike Information Criterion (AIC) values: model 1 which includes scenicness only; model 2 which includes greenspace only and model 3 which includes scenicness and greenspace. AIC values are again transformed to Akaike weights (AICw) in order to facilitate interpretation. This model comparison confirms our earlier findings that the models including scenicness have more explanatory power than the model with only greenspace (Fig. S4).

## Discussion

In conclusion, we use crowdsourced data from the website *Scenic-Or-Not*, where people rate the “scenicness” of geotagged photographs of Great Britain, in order to explore whether a relationship exists between the scenicness of an environment and the reported health of its inhabitants. We also analyze the color composition of each image to help us assess how scenicness differs from greenspace, traditionally measured by green land cover. Crucially, we investigate whether scenicness may offer new insights into geographic differences in reports of health, beyond the variation explained by green land cover alone.

We find that inhabitants of more scenic environments report better health, across urban, suburban and rural areas. This result holds even when taking core socioeconomic indicators of deprivation, such as income, and data on air pollution into account. Importantly, we find that differences in reports of health can be better explained by the scenicness of the local environment than by measurements of greenspace.

Our color analysis also reveals that scenicness does not simply constitute large areas of green. Indeed, we find that the most scenic areas do not contain the most green, but rather contain high proportions of blue, grey and brown. It seems that scenic environments can include large areas of water, open blue skies or mountainous landscapes. Green areas congested with manmade objects such as buildings and roads may deter the enjoyment of greenspace and may cause a decrease in scenicness ratings.

Our analysis of online crowdsourced datasets indicates that the interaction between the built and natural environment and human experience may be more complex than can be explained by studies limited to using objective datasets such as greenspace. We present evidence that aesthetics, as measured by “scenicness,” may play a central role in the environment’s ability to affect our health. These findings provide evidence that the aesthetics of the environment may have a greater practical impact than previously believed. In order to ensure the wellbeing of local inhabitants, it may therefore be valuable to consider the aesthetics of the environment when embarking upon large projects to build new parks, housing developments or highways.

## Methods

### *Scenic-Or-Not* scenic ratings

*Scenic-Or-Not* presents users with random geotagged photographs of Great Britain, most of which have been taken at eye level. Users can rate photographs on an integer scale of 1–10, where 10 indicates “very scenic” and 1 indicates “not scenic”. The *Scenic-Or-Not* dataset comprises images sourced from *Geograph* (http://www.geograph.org.uk/) covering nearly 95% of the 1 km grid squares of Great Britain. Images included in this dataset may have been submitted via *Geograph* at any point since March 2005. Scenicness ratings can be retrieved from *Scenic-Or-Not* for images that have been rated three times or more. As of August 2, 2014, 1,529,927 ratings were available for 212,057 images.

### Data retrieval

We retrieved data on scenicness ratings by accessing the *Scenic-Or-Not* website (http://scenic.mysociety.org) on August 2, 2014. Color naming data was retrieved from *XKCD* (http://blog.xkcd.com/2010/05/03/color-survey-results/) on May 23, 2014. Data on self-reported health from the *2011 Census for England and Wales* was retrieved on July 16, 2014 from *Nomis* (https://www.nomisweb.co.uk/census/2011). Green land cover data was retrieved on June 30, 2014 from *Neighbourhood Statistics* (http://www.neighbourhood.statistics.gov.uk/). Data on the English indices of deprivation was retrieved on July 1, 2014 from *Neighbourhood Statistics* (http://www.neighbourhood.statistics.gov.uk/). Data on the *Rural Urban Classification* was retrieved on June 24, 2014, also from *Neighbourhood Statistics* (http://www.neighbourhood.statistics.gov.uk/). Data on “improved water sources” was retrieved on May 19, 2015 from the World Bank (http://data.worldbank.org/). Data on the quality of drinking water for England and Wales was retrieved on May 19, 2015 from the Drinking Water Inspectorate (http://dwi.defra.gov.uk/). Modeled pollution data was retrieved on May 19, 2015 from the Air Information Resource (http://uk-air.defra.gov.uk/). The boundary data for LSOAs in England was retrieved on December 17, 2013 from the UK Data Service Census Support (http://census.edina.ac.uk/).

### Conditional Auto Regressive (CAR) model

When working with spatial data, it is reasonable to assume that observations in neighboring areas may be more or less alike simply due to their proximity, and hence exhibit autocorrelation^47^. We confirm this by first running a Moran’s I test, which measures whether spatial autocorrelation is present in the data. Due to this autocorrelation, we cannot run a simple linear regression analysis, as spatial dependencies would exist in the error term. Hence, we run our analysis using a conditional autoregressive prior (CAR), as initially proposed by Besag and colleagues^51^,^52^, which captures spatial dependence between neighbors through an adjacency matrix of the areal units.

The CAR model quantifies the spatial relationship in the data by including a conditional distribution in the error term for area i, *e*_*i*_. The conditional distribution of *e*_*i*_ is thus represented as:




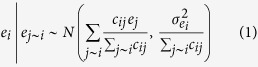




where *e*_*j~i*_ is the *e*_*–i*_ vector including only neighboring areas of i; *e*_*–i*_ is the vector of all the errors terms except for *e*_*–i*_ itself; and *c*_*ij*_ are dependence parameters used to represent the spatial dependence between the areas.

### Akaike weights (AICw)

In order to determine which model best captures variance in the data on poor health, we calculate the Akaike weights (AICws), following the method proposed by Wagenmakers and Farrel^48^, as the AIC values themselves are difficult to interpret on their own. We derive AICws by first identifying the model with the lowest AIC. For each model, we then calculate an AIC difference, by determining the difference between the lowest AIC and the model’s AIC. We next determine the relative likelihood of each model, following the method described in Wagenmakers and Farrel^48^. To determine the AICws we normalize these likelihoods, such that across all models they sum to one. The resulting AICws can be interpreted as the probability of each model given the data.

### Color composition of images

We examine each image from *Scenic-Or-Not* on a per-pixel level, with each pixel being allocated to one of eleven colors that constitute the principal colors in the English vocabulary (black, blue, brown, grey, green, orange, pink, purple, red, white, yellow). As color naming varies from one individual to another^42^, we draw on crowdsourced data generated through an online survey of 1.5 million participants to determine to which color a pixel should be allocated. In this survey, participants were shown an area filled with a random fully-saturated color on both black and white backgrounds, and asked to name the color. These responses were then used to create a list of the dominant color names corresponding to fully saturated RGB (Red, Green, Blue) values. We use this data in order to determine where color boundaries should be drawn: for example, where “brown” ends and “green” begins. The RGB colors are converted to the HSV (Hue, Saturation, Value) color space and each pixel is matched to the closest corresponding color, based on its hue parameter. The nature of the relationship between HSV and RGB space is such that all possible hues are covered by all fully saturated RGB colors. As black, grey and white do not have a defined hue, these color boundaries were determined based on a combination of the levels of “Saturation” and “Value” (Fig. S3).

## Additional Information

**How to cite this article**: Seresinhe, C. I. *et al.* Quantifying the Impact of Scenic Environments on Health. *Sci. Rep.* **5**, 16899; doi: 10.1038/srep16899 (2015).

## Supplementary Material

Supplementary Information

## Figures and Tables

**Table 1:** Predicting poor health with scenicness and greenspace.

	All areas	Urban	Suburban	Rural
Scenicness	−0.008***	−0.007*	−0.005**	−0.012***
Greenspace	−0.008	−0.001	0.020*	0.004
Income Deprivation	1.684***	1.788***	1.416***	1.024***
Employment Deprivation	3.200***	3.114***	3.310***	4.027***
Education Deprivation	0.003***	0.003***	0.003***	0.006***
Housing Deprivation	−0.001***	0.000	−0.001***	−0.001**
Crime	0.009***	−0.004	0.007*	0.013***
Living Deprivation	0.000***	0.001***	0.000*	0.000
AIC	−10938	−1305	−5038	−5458
No of observations	16907	3944	7781	5182

Regression coefficients for CAR models predicting standardized rates of reports of poor health using scenicness and greenspace. In these models, a range of socioeconomic deprivation variables are controlled for. Models are built for England as a whole, and for urban, suburban and rural areas separately. The analysis is carried out at the level of Lower Layer Super Output Areas, such that each data point relates to an area inhabited by roughly 1,600 people. Lower ratings of scenicness are significantly associated with reports of worse health across England as a whole, as well as across urban, suburban and rural areas. However, greenspace only bears a relationship to health in suburban areas, where more greenspace is in fact positively correlated with worse health. *p < 0.05, **p < 0.01, ***p < 0.001.

**Table 2:** Predicting poor health with greenspace only.

	All areas	Urban	Suburban	Rural
Greenspace	−0.019***	−0.011	0.014	−0.008
Income Deprivation	1.696***	1.797***	1.418***	1.024***
Employment Deprivation	3.181***	3.107***	3.301***	4.015***
Education Deprivation	0.003***	0.003***	0.004***	0.006***
Housing Deprivation	−0.001***	0.000	−0.001***	−0.001**
Crime	0.010***	−0.003	0.007*	0.015***
Living Deprivation	0.000***	0.001**	0.000*	−0.001*
AIC	−10904	−1301	−5033	−5443
No of observations	16907	3944	7781	5182

A correlation analysis indicates that scenicness is significantly correlated with greenspace (*τ * = 0.2, *p* < 0.001, *N* = 128,213). We therefore build another four CAR models to predict standardized rates of reports of poor health, using greenspace only. Here, we present the regression coefficients. As in Table 1, models are built for England as a whole, and for urban, suburban and rural areas separately. A range of socioeconomic deprivation variables are controlled for, and the analysis is carried out at the level of Lower Layer Super Output Areas. In this revised model, while less greenspace is significantly associated with reports of worse health, this effect no longer holds when the analysis is broken down into urban, suburban and rural areas. *p < 0.05, **p < 0.01, ***p < 0.001.

**Table 3:** Predicting poor health with scenicness only.

	All areas	Urban	Suburban	Rural
Scenicness	−0.008***	−0.007**	−0.004*	−0.011***
Income Deprivation	1.691***	1.789***	1.404***	1.023***
Employment Deprivation	3.194***	3.113***	3.318***	4.028***
Education Deprivation	0.003***	0.003***	0.004***	0.006***
Housing Deprivation	−0.001***	0.000	−0.001***	−0.001**
Crime	0.009***	−0.004	0.007	0.013***
Living Deprivation	0.000***	0.001**	0.000	0.000
AIC	−10938	−1307	−5035	−5460
No of observations	16907	3944	7781	5182

Regression coefficients for CAR models predicting standardised rates of poor health using scenicness only. As in Tables 1 and 2, models are built for England as a whole, and for urban, suburban and rural areas separately. A range of socioeconomic deprivation variables are controlled for, and the analysis is carried out at the level of Lower Layer Super Output Areas. Again, lower ratings of scenicness are significantly associated with reports of worse health across England as a whole, as well as across urban, suburban and rural areas. As such, the relationship between scenicness and health is similar to that found in the first model presented in Table 1, in which greenspace is included. *p < 0.05, **p < 0.01, ***p < 0.001.
