# Supplementary material for: Quantifying the Impact of Scenic Environments on Health
Source: Sci Rep. 2015 Nov 25;5:16899. doi: 10.1038/srep16899 (PMC4658473; doi:10.1038/srep16899)
Supplement: Supplementary Information [file srep16899-s1.pdf]

Supplementary Information: Quantifying the Impact of Scenic Environments on Health

Chanuki Illushka Seresinhe[1*], Tobias Preis[1], and Helen Susannah Moat[1]

[1] Data Science Lab, Behavioural Science, Warwick Business School,
University of Warwick, Coventry, CV4 7AL, UK

* To whom correspondence should be addressed;
E-mail: C.Seresinhe@warwick.ac.uk

Scenicness ratings and basic characteristics of the photographs

As the degree of the quality of the photographs may in itself affect their scenicness ratings, we also evaluate the relationship between color characteristics of the photographs and their scenic rating. We investigate whether brighter or more color-saturated images correspond with higher ratings. We also investigate whether images with warmer colors, which contain more red, tend to receive higher ratings than images with cooler colors, which contain more blue. We calculate the warmth of an image pixel by extracting its Red, Green and Blue (RGB) values and defining it as warm if the red value exceeds the blue value. We calculate the warmth of an image as the proportion of warm pixels over the total number of pixels in an image.

Figure S2 depicts the relationship between scenicness ratings and the brightness, color saturation and warmth of an image. We build a simple linear regression model to check to what extent higher scenicness ratings can be explained by higher saturation, brightness and warmth values. We find that images with greater color saturation tend to be rated slightly more highly than images with lower color saturation ($\beta = 0.027$, $t(206869) = 74.14$, $p < 0.001$). However, it is unclear whether saturation is a property of the scenic areas themselves or of the photographs. For instance, the sample of images with high scenicness ratings and low scenicness ratings presented in Fig. 1 suggests that images with higher ratings may contain fewer low-saturation grey manmade structures. Furthermore, although the linear regression analysis suggests that both brightness ($\beta = 0.010$, $t(206869) = 23.02$, $p < 0.001$) and warmth ($\beta = 0.004$, $t(206869) = 18.74$, $p < 0.001$) significantly increase with scenicness ratings, visual inspection suggests brightness and warmth do not have a simple linear relationship with scenicness, where warmth in particular appears to be

highest for pictures with a medium scenic rating of around 5 or 6, and thus do not steadily increase or decrease with each consecutive scenic rating (Fig. S2).

Analyzing pollutants using Principal Component Analysis (PCA)

Strong collinearity between predictor variables can make it impossible to identify which predictor variable best explains the dependent variable in a regression model. We therefore investigate to which extent collinearity exists between modeled estimates of concentrations of the following pollutants: sulphur dioxide (SO_2), oxides of nitrogen (NOx), particles and fine particles (PM_{10} and $PM_{2.5}$), benzene (C_6H_6), carbon monoxide (CO) and ozone (O_3). Following the method proposed by Belsley, Kuh and Welsch[1] , we find that high collinearity (condition number: 285.03) exists between six of the pollutant variables: sulphur dioxide (SO_2), oxides of nitrogen (NOx), particles and fine particles (PM_{10} and $PM_{2.5}$), benzene (C_6H_6) and carbon monoxide (CO). We therefore reduce these six correlated variables into three uncorrelated variables using Principal Component Analysis (PCA). The three PCA variables chosen each explain more than 5% of the variance of the original variables, and cumulatively account for 95.74% of the variance of the original variables.

Table S1 | Results of AIC Analysis for varying models. Akaike weights (AICw) can be interpreted as the probability of the model given the data. Further details on how a model's AICw is calculated can be found in the Methods section. In all cases, there is more evidence for the models that include scenicness than for the model with only greenspace.

Urbanity	Model	AIC	AICd	AICw
All areas	Scenicness and Greenspace	-10938	0	0.500
	Greenspace only	-10904	34	0.000
	Scenicness only	-10938	0	0.500
Urban	Scenicness and Greenspace	-1305	2	0.260
	Greenspace only	-1301	6	0.032
	Scenicness only	-1307	0	0.708
Suburban	Scenicness and Greenspace	-5038	0	0.771
	Greenspace only	-5033	5	0.057
	Scenicness only	-5035	3	0.172
Rural	Scenicness and Greenspace	-5458	0	1.000
	Greenspace only	-5443	15	0.000
	Scenicness only	-5038	420	0.000

Table S2 | Predicting poor health with scenicness and greenspace. Regression coefficients for CAR models predicting standardized rates of reports of poor health using scenicness and greenspace. In these models, a range of socioeconomic deprivation variables are controlled for. We also include measures calculated using Principal Component Analysis (PCA), which represent concentrations of the following pollutants: sulphur dioxide (SO_2), oxides of nitrogen (NOx), particles and fine particles (PM_{10} and $PM_{2.5}$), benzene (C_6H_6) and carbon monoxide (CO). See Supplementary Information for more information on how these PCA variables have been calculated. We also control for concentrations of ozone (O3). Models are built for England as a whole, and for urban, suburban and rural areas separately. The analysis is carried out at the level of Lower Layer Super Output Areas, such that each data point relates to an area inhabited by roughly 1,600 people. Lower ratings of scenicness are significantly associated with reports of worse health across England as a whole, as well as across urban, suburban and rural areas. Across England as a whole, we also find that more greenspace is associated with worse health. This relationship however does not hold in urban and rural areas when they are analyzed separately.

	All areas	Urban	Suburban	Rural
Scenicness	-0.006 ***	-0.008 **	-0.004 *	-0.008 **
Greenspace	0.037 ***	-0.006	0.05 ***	0.025
Income Deprivation	1.581 ***	1.915 ***	1.382 ***	1.115 ***
Employment Deprivation	3.189 ***	2.712 ***	3.264 ***	3.977 ***
Education Deprivation	0.004 ***	0.003 ***	0.004 ***	0.006 ***
Housing Deprivation	-0.001 ***	0.000	-0.001 ***	0.000 *
Crime	0.003	0.001	0.005	0.001
Living Deprivation	0.000	0.001 *	0.000	0.000
PCA 1	-0.002 *	0.009 **	-0.001	-0.013 ***
PCA 2	-0.017 ***	-0.009	-0.02 ***	-0.013 ***
PCA 3	-0.013 ***	-0.007	-0.016 ***	-0.007
Ozone (O_3)	-0.018 ***	-0.069 ***	-0.018 ***	-0.01 ***
AIC	-11293	-1358	-5192	-5563
No of observations	16907	3944	7781	5182

* $p < 0.05$, ** $p < 0.01$, *** $p < 0.001$

Table S3 | Predicting poor health with greenspace only. Regression coefficients for CAR models predicting standardized rates of poor health using greenspace only. As in Table 1, models are built for England as a whole, and for urban, suburban and rural areas separately. We also control for the following pollutants: sulphur dioxide (SO_2), oxides of nitrogen (NOx), particles and fine particles (PM_{10} and $PM_{2.5}$), benzene (C_6H_6), carbon monoxide (CO) and ozone (O3), using the measures introduced in Table S2. A range of socioeconomic deprivation variables are controlled for, and the analysis is carried out at the level of Lower Layer Super Output Area. In this model, we find that more greenspace is significantly associated with reports of worse health across England as a whole. However, this effect does not hold in urban or rural areas when they are analyzed separately.

	All areas	Urban	Suburban	Rural
Greenspace	0.03 ***	-0.016	0.046 ***	0.019
Income Deprivation	1.583 ***	1.921 ***	1.382 ***	1.116 ***
Employment Deprivation	3.186 ***	2.71 ***	3.261 ***	3.982 ***
Education Deprivation	0.004 ***	0.003 ***	0.004 ***	0.006 ***
Housing Deprivation	-0.001 ***	0.000	-0.001 ***	0.000 *
Crime	0.003	0.002	0.005	0.001
Living Deprivation	0.000	0.001 *	0.000	0.000
PCA 1	-0.003 **	0.008 **	-0.001	-0.015 ***
PCA 2	-0.016 ***	-0.008	-0.02 ***	-0.013 ***
PCA 3	-0.013 ***	-0.007	-0.016 ***	-0.007
Ozone (O_3)	-0.018 ***	-0.069 ***	-0.018 ***	-0.01 ***
AIC	-11273	-1353	-5189	-5557
No of observations	16907	3944	7781	5182

* $p < 0.05$, ** $p < 0.01$, *** $p < 0.001$

Table S4 | Predicting poor health with scenicness only. Regression coefficients for CAR models predicting standardized rates of poor health using scenicness only. As in Table 1, models are built for England as a whole, and for urban, suburban and rural areas separately. We also control for the following pollutants: sulphur dioxide (SO_2), oxides of nitrogen (NOx), particles and fine particles (PM_{10} and $PM_{2.5}$), benzene (C_6H_6), carbon monoxide (CO) and ozone (O3), using the measures introduced in Table S2. A range of socioeconomic deprivation variables are controlled for, and the analysis is carried out at the level of Lower Layer Super Output Area. Again, lower ratings of scenicness are significantly associated with reports of worse health across England as a whole. While lower ratings of scenicness are significantly associated with reports of worse health across urban and rural areas, this relationship does not hold in suburban areas.

	All areas	Urban	Suburban	Rural
Scenicness	-0.005 ***	-0.008 **	-0.002	-0.007 *
Income Deprivation	1.578 ***	1.917 ***	1.367 ***	1.1 ***
Employment Deprivation	3.189 ***	2.712 ***	3.268 ***	3.988 ***
Education Deprivation	0.004 ***	0.003 ***	0.004 ***	0.006 ***
Housing Deprivation	0.000 **	0.000	-0.001 *	0.000
Crime	0.003	0.001	0.007	0.002
Living Deprivation	0.000	0.001 *	0.000	0.000
PCA 1	-0.002	0.009 **	0.000	-0.013 ***
PCA 2	-0.014 ***	-0.009	-0.017 ***	-0.012 ***
PCA 3	-0.01 ***	-0.008	-0.012 ***	-0.005
Ozone (O_3)	-0.019 ***	-0.068 ***	-0.02 ***	-0.01 ***
AIC	-11268	-1359	-5166	-5562
No of observations	16907	3944	7781	5182

* $p < 0.05$, ** $p < 0.01$, *** $p < 0.001$

Table S5 | Results of AIC Analysis for varying models which additionally include the pollutant variables sulphur dioxide (SO_2), oxides of nitrogen (NOx), particles and fine particles (PM_{10} and $PM_{2.5}$), benzene (C_6H_6), carbon monoxide (CO) and ozone (O3). Akaike weights (AICw) can be interpreted as the probability of the model given the data. Further details on how a model's AICw is calculated can be found in the Methods section. In all cases, the models that include scenicness perform better than the model with only greenspace.

Urbanity	Model	AIC	AICd	AICw
All areas	Scenicness and Greenspace	-10938	0	0.500
	Greenspace only	-10904	34	0.000
	Scenicness only	-10938	0	0.500
Urban	Scenicness and Greenspace	-1305	2	0.260
	Greenspace only	-1301	6	0.032
	Scenicness only	-1307	0	0.708
Suburban	Scenicness and Greenspace	-5038	0	0.771
	Greenspace only	-5033	5	0.057
	Scenicness only	-5035	3	0.172
Rural	Scenicness and Greenspace	-5458	0	1.000
	Greenspace only	-5443	15	0.000
	Scenicness only	-5038	420	0.000

FIGURES

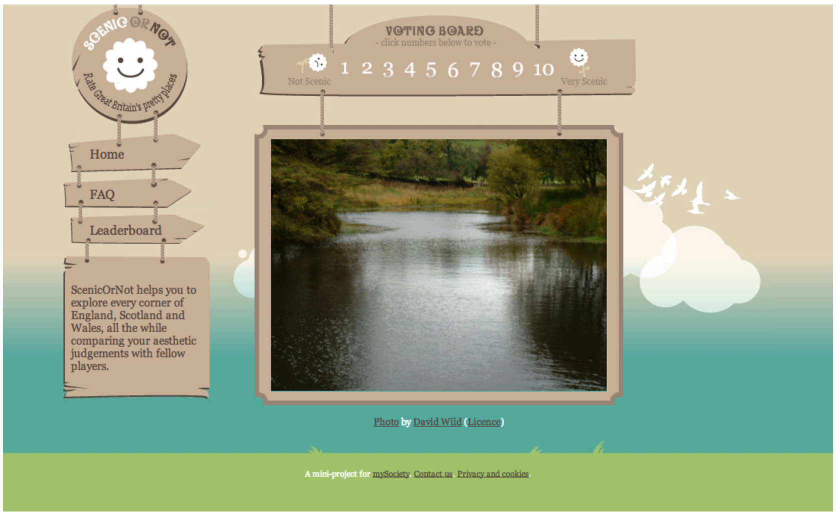

Figure S1 | **The *Scenic-Or-Not* voting screen.** *Scenic-Or-Not* presents users with random geotagged photographs of Great Britain, which visitors can rate on an integer scale of 1 – 10 (10 indicating "very scenic" and 1 indicating "not scenic"). The dataset contains 217,000 images, sourced from *Geograph* (http://www.geograph.org.uk), covering nearly 95% of the 1 km grid squares in Great Britain. Scenic image by David Wild (http://www.geograph.org.uk/photo/35940). Image is licensed for reuse under the Creative Commons Attribution-Share Alike 2.0 Generic License. To view a copy of this licence, visit http://creativecommons.org/licenses/by-sa/2.0/

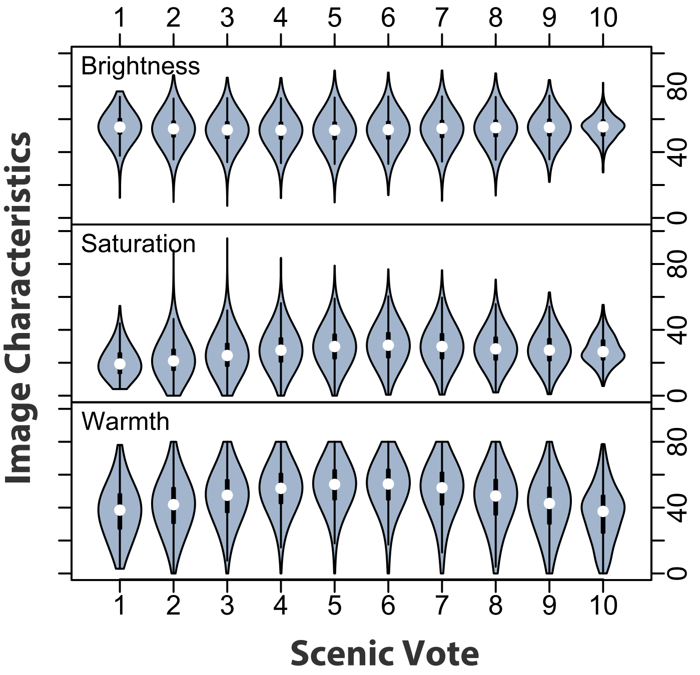

Figure S2 | Image characteristics of photographs on *Scenic-Or-Not*. We find that images with greater color saturation tend to be rated slightly more highly than images with lower color saturation ($\beta = 0.027$, $t(206869) = 74.14$, $p < 0.001$). However, it is unclear whether saturation is a property of the scenic areas themselves or of the photographs. For instance, the sample of images with high scenicness ratings and low scenicness ratings presented in Fig. 1 suggests that images with higher ratings may contain fewer low-saturation grey manmade structures. Furthermore, although a linear regression analysis suggests that both brightness ($\beta = 0.010$, $t(206869) = 23.02$, $p < 0.001$) and warmth ($\beta = 0.004$, $t(206869) = 18.74$, $p < 0.001$) significantly increase with scenicness ratings, visual inspection suggests brightness and warmth do not have a simple linear relationship with scenicness.

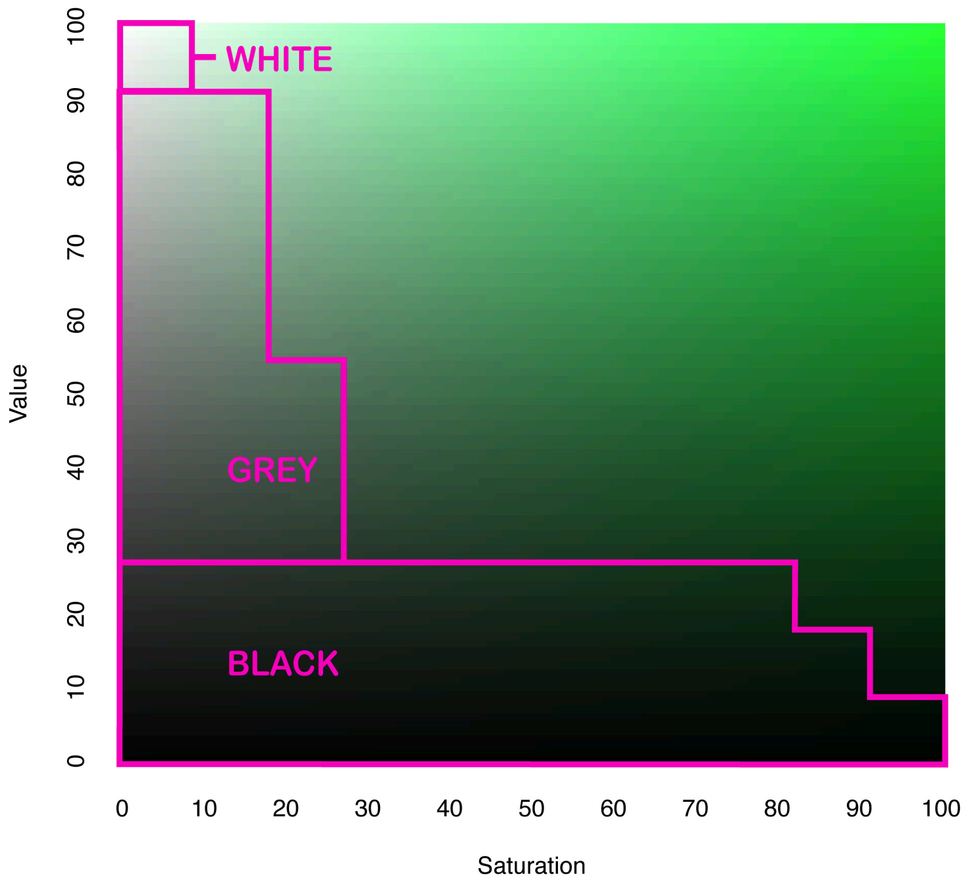

Figure S3 | Allocating black, grey and white based on value and saturation. Here, the hue has been set to one color (green) in order to illustrate where the boundaries of black, grey and white are set. At the borders, the color may appear to be more or less grey, as well as more or less black, depending on the hue, so the boundaries are chosen as a best-fit compromise over the entire range of hue values.

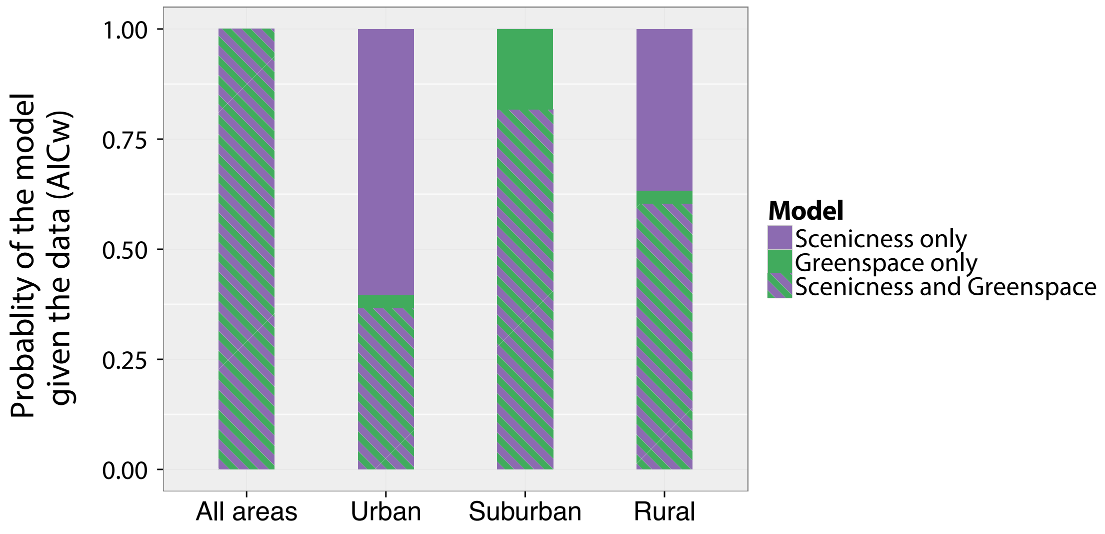

Figure S4 | Model comparison using for varying models which additionally include the pollutant variables: sulphur dioxide (SO_2), oxides of nitrogen (NOx), particles and fine particles (PM_{10} and $PM_{2.5}$), benzene (C_6H_6), carbon monoxide (CO), ozone (O3). We investigate to what extent geographic differences in health can be explained by scenicness and greenspace, by creating CAR models in which we also control for socioeconomic deprivation using data from the 2010 English Indices of Deprivation. We also control for the following pollutants: sulphur dioxide (SO_2), oxides of nitrogen (NOx), particles and fine particles (PM_{10} and $PM_{2.5}$), benzene (C_6H_6), carbon monoxide (CO), ozone (O3) using the measures introduced in Table S2. To determine which model provides the best fit for predicting poor health, we calculate Akaike weights (AICw) which can be used to interpret the probability of each model given the data. Further details on how a model's AICw is calculated can be found in the Methods section. In all cases, we find that models that include scenicness (denoted by the color purple or by purple and green stripes) perform better than the model with only greenspace (denoted by the color green).

References

1. Belsley, D. A., Kuh, E. & Welsch, R. E. Detecting Influential Observations and Outliers 6-84 (John Wiley & Sons, Inc., 1980).
